# From CAR-T to CAAR-T: redefining targeting strategies in B-cell malignancies through idiotype-directed cellular therapy

**DOI:** 10.3389/fimmu.2026.1842236

**Published:** 2026-05-18

**Authors:** Tomasz Buczek, Aleksandra Butrym

**Affiliations:** 1 Department of Hematology and Oncology, Wroclaw Medical University Branch in Walbrzych, Walbrzych, Poland; 2 Oncology Support Centre for Clinical Trials, Alfred Sokolowski Specialist Hospital in Walbrzych, Walbrzych, Poland; 3 Clinic of Hematology and Oncology, Wroclaw Medical University, Walbrzych, Poland

**Keywords:** B-cell malignancies, B-cell receptor, CAAR-T, CAR-T, chronic lymphocytic leukemia, clonal targeting, indolent lymphoma, precision immunotherapy

## Abstract

Chimeric antigen receptor T-cell (CAR-T) therapy has transformed the management of selected hematologic malignancies, particularly relapsed or refractory large B-cell lymphoma, B-cell acute lymphoblastic leukemia, and multiple myeloma. However, currently approved CAR-T strategies largely rely on lineage-associated or differentiation antigens, such as CD19 or BCMA, and therefore do not selectively distinguish malignant B cells from their normal counterparts. This limitation contributes to on-target, off-tumor toxicity, including B-cell aplasia, hypogammaglobulinemia, infectious complications, and prolonged immune dysfunction. In addition, CAR-T therapy remains associated with cytokine release syndrome, immune effector cell-associated neurotoxicity syndrome, immune effector cell-associated hematotoxicity, manufacturing complexity, and variable efficacy across disease entities, particularly in chronic lymphocytic leukemia. Chimeric autoantibody receptor T-cell (CAAR-T) therapy represents a conceptually distinct approach in which engineered T cells are designed to recognize disease-defining immunoglobulin structures, including surface immunoglobulin or B-cell receptor idiotypes. This strategy has been most extensively explored in autoimmune diseases, where CAAR-T cells can selectively eliminate autoreactive B-cell populations while sparing the broader B-cell compartment. Its application in B-cell malignancies remains largely hypothetical, but the biological principle is attractive because many B-cell neoplasms are defined by clonotypic immunoglobulin rearrangements. In this review, we provide a disease-specific and translationally oriented assessment of idiotype-directed CAAR-T therapy in B-cell malignancies. We summarize the current evidence supporting CAAR-T biology, critically evaluate its potential in chronic lymphocytic leukemia, indolent lymphomas, multiple myeloma, and minimal residual disease, and discuss key biological, economic, manufacturing, and regulatory barriers. At present, CAAR-T should not be viewed as a near-term replacement for approved CAR-T therapies, but rather as a hypothesis-generating precision platform requiring rigorous disease-specific validation.

## Introduction

Adoptive cellular therapies based on genetically modified T lymphocytes have reshaped the therapeutic landscape of hematologic oncology (1–4). Among them, chimeric antigen receptor T-cell (CAR-T) therapy has achieved the greatest clinical maturity and is now incorporated into the treatment algorithms of several relapsed or refractory B-cell malignancies. By redirecting autologous or allogeneic T cells toward predefined surface antigens independently of major histocompatibility complex presentation, CAR-T therapy can induce deep and durable remissions in patients with otherwise limited therapeutic options (3, 4).

The clinical adoption of CAR-T therapy has been supported by pivotal trials across several disease entities. In aggressive large B-cell lymphoma, ZUMA-1 established the activity of axicabtagene ciloleucel in refractory disease (5, 6). In indolent lymphoma, tisagenlecleucel also demonstrated activity in relapsed or refractory follicular lymphoma (7), while subsequent randomized phase III trials such as ZUMA-7 demonstrated the superiority of axicabtagene ciloleucel over standard second-line therapy in patients with early relapsed or refractory large B-cell lymphoma (8). Other studies, including TRANSFORM and BELINDA, further defined the role of different CD19-directed CAR-T products in relapsed or refractory large B-cell lymphoma (9, 10). In B-cell acute lymphoblastic leukemia, tisagenlecleucel demonstrated substantial activity in pediatric and young adult patients (11), while in multiple myeloma, BCMA-directed products such as idecabtagene vicleucel and ciltacabtagene autoleucel have produced deep responses in heavily pretreated patients (12, 13). Collectively, these studies established CAR-T therapy as one of the most important therapeutic advances in modern hemato-oncology.

Despite these achievements, the current CAR-T paradigm has important limitations. Most approved CAR-T products target lineage-associated or differentiation antigens, most prominently CD19 in B-cell malignancies and BCMA in multiple myeloma (3, 12, 13). These antigens are not tumor-specific. Consequently, therapeutic activity is intrinsically linked to collateral depletion of normal antigen-expressing cells. In CD19-directed therapy, this results in B-cell aplasia, hypogammaglobulinemia, increased susceptibility to infection, and the need for long-term immune monitoring (14, 15). In BCMA-directed therapy, plasma-cell targeting may similarly affect humoral immunity. These effects are clinically manageable in many patients, but they represent a meaningful form of treatment-induced immunodeficiency, particularly in diseases with prolonged natural histories.

CAR-T therapy is also associated with acute and delayed toxicities. Cytokine release syndrome (CRS) and immune effector cell-associated neurotoxicity syndrome (ICANS) remain central safety concerns and require specialized clinical infrastructure (16, 17). More recently, immune effector cell-associated hematotoxicity (ICAHT) has emerged as a distinct and clinically relevant complication characterized by prolonged cytopenias, delayed hematopoietic recovery, infectious risk, transfusion dependence, and potential contribution to non-relapse morbidity (18). These toxicities underscore that the therapeutic success of CAR-T therapy is accompanied by significant biological and logistical complexity.

Efficacy also varies substantially by disease entity. In diffuse large B-cell lymphoma and B-cell acute lymphoblastic leukemia, CAR-T therapy has produced durable remissions in a clinically meaningful proportion of patients. In chronic lymphocytic leukemia (CLL), however, responses have been less consistent, probably reflecting intrinsic T-cell dysfunction, prior treatment exposure, immune exhaustion, and a profoundly immunosuppressive tumor microenvironment (19–22). These observations have stimulated interest in more selective approaches that could distinguish malignant B-cell clones from the normal B-cell compartment and possibly reduce toxicity without sacrificing efficacy.

Chimeric autoantibody receptor T-cell (CAAR-T) therapy represents one such strategy. Unlike conventional CAR-T cells, which recognize a shared surface antigen through an antibody-derived binding domain, CAAR-T cells are designed to recognize immunoglobulin structures expressed by pathogenic B-cell populations. In autoimmune disease, this can be achieved by expressing an autoantigen as the extracellular recognition domain, allowing selective elimination of autoreactive B cells (23–27). In B-cell malignancies, an analogous approach could theoretically target clonotypic surface immunoglobulin or B-cell receptor (BCR) idiotypes expressed by malignant clones.

The rationale is biologically compelling. B-cell malignancies arise from clonal populations defined by unique immunoglobulin gene rearrangements. These rearrangements generate BCRs with clonotypic idiotypes that may function as tumor-specific markers (28–31). Targeting the idiotype could, in principle, allow destruction of malignant B cells while sparing normal B cells with unrelated antigen receptors. This would represent a shift from lineage-level targeting to clone-level targeting.

However, this concept remains largely unproven in hemato-oncology. Most experimental and early translational experience with CAAR-T therapy comes from autoimmune disease rather than cancer. B-cell malignancies present additional challenges, including clonal evolution, subclonal heterogeneity, variable surface immunoglobulin expression, antigen modulation, soluble immunoglobulin interference, immune escape, and manufacturing complexity (35–40). Therefore, any discussion of CAAR-T in cancer must distinguish clearly between biological plausibility and clinical validation.

**Figure 1:**
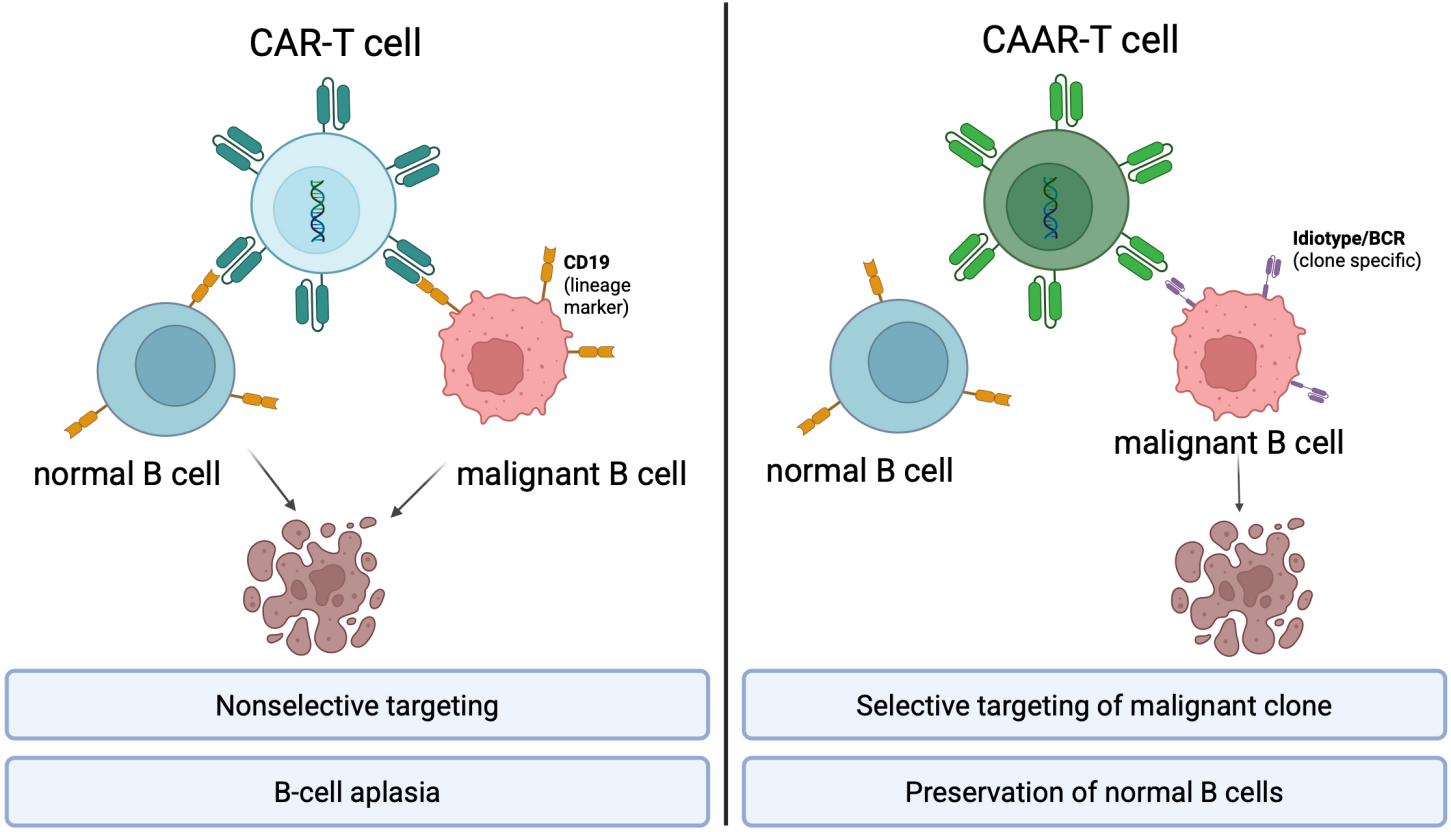
Annotated conceptual comparison of CAR-T and CAAR-T targeting strategies. CAR-T cells recognize lineage-associated antigens (e.g., CD19), leading to nonselective depletion of both malignant and normal B cells and resulting in B-cell aplasia (3, 5, 14, 15). In contrast, CAAR-T cells bind clonotypic B-cell receptors (idiotypes), enabling selective elimination of malignant clones while preserving normal B cells (23–27). Key advantages and limitations of each approach are highlighted (34, 35, 39–45). Created in BioRender.com by Buczek, T. (2026) https://BioRender.com/ukmc9o3.

While previous reviews have focused broadly on CAR-T therapy or on CAAR-T applications in autoimmune disease, this review provides a disease-specific and translationally oriented evaluation of CAAR-T in B-cell malignancies. We critically assess the biological feasibility, potential clinical niches, and practical limitations of idiotype-directed targeting, with particular attention to CLL, indolent lymphomas, multiple myeloma, and minimal residual disease. Rather than proposing CAAR-T as an immediate alternative to approved CAR-T therapies, we frame it as a precision immunotherapy platform requiring rigorous preclinical and clinical validation. These fundamental differences between CAR-T and CAAR-T targeting strategies are illustrated in **Figure 1**.

## Conceptual distinction between CAR-T and CAAR-T therapy

CAR-T and CAAR-T therapies share a common cellular engineering principle: both redirect T cells through synthetic receptors that couple extracellular recognition to intracellular activation (3, 4). However, they differ fundamentally in the nature of the target and in the biological logic of recognition.

Conventional CAR-T cells are typically designed to recognize a predefined surface antigen expressed across a malignant lineage or differentiation state. CD19-directed CAR-T cells recognize CD19 on malignant and normal B cells, while BCMA-directed CAR-T cells recognize BCMA on malignant plasma cells and normal plasma-cell populations (5, 12, 13). This strategy is powerful because the target is shared across many patients and can support standardized product development. However, the price of this scalability is limited specificity. The target defines a cellular compartment rather than an individual malignant clone.

CAAR-T therapy, in contrast, is based on selective recognition of pathogenic immunoglobulin structures. In autoimmune disease, the CAAR extracellular domain may contain an autoantigen that binds B cells expressing autoreactive BCRs (23–27). In a malignancy-directed idiotype approach, the target would be the clonotypic BCR or surface immunoglobulin expressed by the malignant clone. This distinction is central: CAAR-T does not merely target a cell lineage; it seeks to target the molecular identity of the disease-driving B-cell population.

The terminology requires precision. The BCR is a membrane-bound immunoglobulin complex associated with signaling components CD79A and CD79B (28–31). The idiotype refers to antigenic determinants within the variable region of an immunoglobulin molecule, particularly within complementarity-determining regions. Surface immunoglobulin may serve as an accessible marker of the malignant clone, whereas secreted immunoglobulin or paraprotein may act as soluble antigen and potentially interfere with receptor engagement (38). Therefore, the feasibility of idiotype-directed CAAR-T depends not only on clonality but also on surface accessibility, antigen density, receptor stability, and the ratio between membrane-bound and soluble immunoglobulin.

This distinction is particularly important when translating CAAR-T from autoimmune disease to cancer. In autoimmune disease, the therapeutic goal is deletion of autoreactive B cells producing pathogenic antibodies. In cancer, the target is a malignant population capable of proliferation, immune evasion, clonal evolution, and antigenic escape. Therefore, proof-of-concept in autoimmunity establishes biological feasibility of selective B-cell targeting, but does not by itself demonstrate antitumor efficacy (23–27, 40).

## Biological rationale for idiotype-directed targeting in B-cell malignancies

The defining biological feature of B-cell malignancies is clonality. Malignant B cells originate from a single transformed cell and typically retain immunoglobulin gene rearrangements that define the clonal population. These rearrangements produce unique BCR sequences and idiotypes that may serve as highly specific molecular identifiers (28–31). In principle, this makes the BCR idiotype an attractive therapeutic target.

The idea of targeting tumor idiotypes is not new. Idiotype vaccines were investigated decades ago, particularly in follicular lymphoma, with the aim of inducing patient-specific immune responses against tumor-derived immunoglobulin (32–34). Although the biological rationale was strong, clinical efficacy was inconsistent and generally insufficient to support widespread adoption (32, 33). Several factors likely contributed to this limitation, including weak immunogenicity, inadequate T-cell help, immune tolerance, tumor-induced immunosuppression, and the inability to generate durable cytotoxic responses.

CAAR-T therapy revisits this concept through a different mechanism. Instead of relying on endogenous immune priming, CAAR-T cells provide an engineered effector population capable of direct recognition and cytotoxicity. This could theoretically overcome some limitations of idiotype vaccination by supplying an active cellular effector rather than merely attempting to induce one. The strategy may be especially relevant in diseases where BCR signaling contributes to pathogenesis, such as CLL and indolent lymphomas.

In CLL, BCR signaling is central to disease biology (29–31, 36). The presence of stereotyped BCR subsets suggests that antigen selection contributes to leukemogenesis and disease behavior. In indolent lymphomas, including follicular lymphoma, tumor immunoglobulin expression has long been recognized as a tumor-specific marker (32, 33). These features support the conceptual rationale for idiotype-directed targeting.

However, a target being tumor-specific does not automatically make it therapeutically robust. For CAAR-T therapy to be effective, several conditions must be met. The target idiotype must be present on most or all malignant cells, sufficiently expressed on the cell surface, stable over time, accessible to engineered T cells, and not excessively neutralized by soluble immunoglobulin. Moreover, loss of the targeted idiotype must either be biologically unlikely or associated with reduced tumor fitness. These requirements are demanding and may differ substantially between disease entities.

## Biological obstacles to idiotype-directed CAAR-T therapy

Despite its conceptual appeal, idiotype-directed targeting faces several biological obstacles that may limit its therapeutic robustness.

First, B-cell malignancies are genetically and phenotypically heterogeneous (35, 40). Even when a dominant clonotypic immunoglobulin rearrangement is identifiable, subclonal architecture may evolve over time or under treatment pressure (35, 40). A CAAR-T product directed against a single idiotype could eliminate the dominant clone while sparing minor subclones with altered or absent target expression. This may be particularly relevant in diseases with branched evolution or late-stage treatment resistance.

Second, BCR and surface immunoglobulin expression are variable (28–31, 36, 37). Some malignant B cells express abundant surface immunoglobulin, whereas others show low or functionally uncoupled BCR expression. In CLL, BCR signaling competence and receptor internalization may differ across subsets (36, 37). In multiple myeloma, mature plasma cells often have limited surface immunoglobulin expression, making idiotype targeting less practical despite the presence of a secreted monoclonal protein (38).

Third, antigen modulation may impair efficacy. Engagement of the BCR may induce internalization, downregulation, or altered signaling. For receptor-directed cellular therapy, this creates uncertainty about sustained target availability. If target engagement rapidly reduces surface expression, CAAR-T activation and serial killing may be compromised.

Fourth, soluble immunoglobulin may function as an antigen sink. This issue is especially relevant in plasma-cell disorders and some lymphoplasmacytic malignancies. High concentrations of soluble tumor-associated proteins, including paraprotein in plasma-cell disorders, could theoretically interfere with receptor engagement, analogous to soluble antigen-sink phenomena described for BCMA-directed immune therapies (38).

Fifth, immune escape remains a major concern. Tumors subjected to highly specific selective pressure may escape through target loss, idiotype variation, reduced antigen density, lineage plasticity, or outgrowth of pre-existing antigen-negative subclones. Historical experience with anti-idiotype therapies in lymphoma demonstrated the emergence of idiotype variants, underscoring the need to consider escape as an expected biological risk rather than a theoretical possibility (39, 40). The key biological challenges associated with idiotype-directed CAAR-T therapy, along with their potential clinical implications and mitigation strategies, are summarized in **Table 1**.

**Table 1:** Biological challenges associated with idiotype-directed CAAR-T therapy in B-cell malignancies.

Challenge	Mechanism	Disease context most relevant	Clinical implication	Potential mitigation strategy
Clonal heterogeneity	Coexistence of subclones with variable BCR/idiotype features	CLL, follicular lymphoma, advanced lymphomas, multiple myeloma	Persistence or relapse from untargeted subclones	Multi-idiotype targeting; combination with lineage-directed therapy; MRD setting
BCR modulation	Downregulation or internalization after receptor engagement	CLL, indolent lymphomas	Reduced target availability and impaired serial killing	Preclinical testing of receptor stability and antigen density
Low surface immunoglobulin	Reduced membrane target expression	Multiple myeloma, plasmablastic differentiation	Limited applicability despite clonotypic immunoglobulin	Patient selection based on surface Ig expression
Soluble immunoglobulin	Circulating paraprotein or free immunoglobulin binds CAAR domain	Multiple myeloma, lymphoplasmacytic lymphoma	Antigen sink, impaired tumor-cell engagement	Affinity optimization; debulking; focus on low-burden disease
Idiotype evolution	Selection of escape variants under immune pressure	Follicular lymphoma, CLL	Antigen-negative relapse	Longitudinal sequencing; dual targeting; adaptive receptor platforms
Immune microenvironment	T-cell exhaustion, suppressive cytokines, inhibitory ligands	CLL, heavily pretreated disease	Reduced CAAR-T expansion and persistence	Combination strategies; optimized manufacturing; earlier disease settings

Idiotype-directed targeting introduces several disease-specific biological limitations that may affect therapeutic efficacy. These include clonal heterogeneity, variability in B-cell receptor (BCR) expression, antigen modulation, the presence of soluble immunoglobulin acting as an antigen sink, and the risk of immune escape through idiotype evolution. The table summarizes key mechanisms, disease contexts in which these challenges are most relevant, their potential clinical implications, and possible mitigation strategies. BCR, B-cell receptor; CLL, chronic lymphocytic leukemia; MRD, minimal residual disease. Based on concepts and evidence discussed in references (28–31, 35–40).

## CAR-T therapy in hematologic malignancies: achievements and limitations

The success of CAR-T therapy provides the clinical foundation for considering more refined cellular immunotherapy strategies. CD19-directed CAR-T therapy has demonstrated major activity in relapsed or refractory aggressive B-cell lymphomas, B-cell acute lymphoblastic leukemia, mantle cell lymphoma, and indolent lymphomas (5–11). BCMA-directed CAR-T therapy has similarly changed expectations for heavily pretreated multiple myeloma (12, 13). These advances confirm that engineered T cells can mediate potent antitumor activity in hematologic malignancies.

However, the limitations of current CAR-T therapy are directly relevant to the rationale for CAAR-T. First, lineage-antigen targeting is inherently nonselective. CD19-directed CAR-T cells eliminate both malignant and normal CD19-positive B cells, resulting in B-cell aplasia and hypogammaglobulinemia. Although immunoglobulin replacement and antimicrobial prophylaxis can mitigate risk, prolonged immune impairment remains clinically meaningful (14, 15, 46).

Second, CAR-T therapy is associated with acute inflammatory toxicity. CRS reflects systemic immune activation and cytokine release following CAR-T engagement and expansion (16). ICANS represents a distinct neurotoxicity syndrome that can range from mild confusion to severe encephalopathy, seizures, cerebral edema, or death (17). These toxicities require trained multidisciplinary teams, standardized grading, intensive monitoring, and access to agents such as tocilizumab and corticosteroids.

Third, delayed hematologic toxicity is increasingly recognized as a major determinant of post-CAR-T morbidity. ICAHT encompasses early, prolonged, or late cytopenias after immune effector cell therapy (18). Clinically, it may manifest as neutropenia, anemia, thrombocytopenia, transfusion dependence, infection risk, delayed immune reconstitution, and prolonged hospitalization. Its pathogenesis is multifactorial and may include baseline marrow reserve, prior therapies, systemic inflammation, macrophage activation, endothelial dysfunction, and disruption of the bone marrow microenvironment. The recognition of ICAHT broadens the toxicity framework beyond CRS and ICANS and highlights the need for strategies that reduce inflammatory and hematopoietic burden.

Fourth, CAR-T efficacy is not uniform across diseases. CLL has been particularly challenging. Despite expression of CD19, clinical responses have historically been less frequent and less durable than in aggressive lymphoma or acute lymphoblastic leukemia. CLL is characterized by T-cell exhaustion, altered immune synapse formation, suppressive microenvironmental signals, and frequent prior exposure to therapies that may impair T-cell fitness (19–22). These factors may limit the performance of autologous cellular products.

Finally, CAR-T therapy remains logistically and economically complex. Autologous manufacturing requires leukapheresis, ex vivo activation, genetic modification, expansion, release testing, lymphodepletion, and specialized infusion infrastructure (41–45). Although increasingly standardized, these processes remain expensive, time-sensitive, and difficult to deliver equitably. Any next-generation personalized cellular therapy, including CAAR-T, must therefore be evaluated not only for biological elegance but also for operational feasibility. The principal conceptual and translational differences between conventional CAR-T and idiotype-directed CAAR-T approaches are summarized in **Table 2**.

**Table 2:** Conceptual comparison of conventional CAR-T and malignancy-directed CAAR-T therapy.

Feature	Conventional CAR-T	Idiotype-directed CAAR-T
Main target	Shared lineage or differentiation antigen such as CD19 or BCMA	Clonotypic BCR/surface immunoglobulin idiotype
Level of specificity	Lineage-level or differentiation-stage specificity	Clone-level specificity
Product generalizability	Same target can be used across many patients	Likely patient-specific or subgroup-specific
Current clinical evidence in cancer	Strong; multiple approved products and pivotal trials	No established clinical evidence in B-cell malignancies
Main therapeutic advantage	Potent activity against antigen-positive malignant cells	Potential sparing of normal B cells and reduced off-tumor depletion
Main biological limitation	On-target, off-tumor toxicity; antigen loss	Clonal heterogeneity; idiotype escape; surface Ig variability
B-cell aplasia	Expected with CD19-directed therapy	Theoretically avoidable if targeting is truly clone-specific
Toxicity profile	CRS, ICANS, ICAHT, infections, hypogammaglobulinemia	Not defined in malignancy; potential inflammatory and antigen-sink risks
Manufacturing	Increasingly standardized autologous or allogeneic platforms	Highly individualized design and validation may be required
Scalability	Moderate to improving	Currently uncertain and potentially limited
Regulatory maturity	Approved and guideline incorporated	Exploratory; regulatory pathway uncertain
Suitability for bulky disease	Demonstrated in selected malignancies	Uncertain; may be limited by antigen burden and heterogeneity
Suitability for MRD	Possible but not the dominant current use	Potentially attractive due to low disease burden and high specificity

Conventional CAR-T therapy targets shared lineage or differentiation antigens, resulting in broad antitumor activity but also on-target, off-tumor effects such as B-cell aplasia. In contrast, CAAR-T therapy is designed to recognize clonotypic immunoglobulin structures, enabling theoretically more selective targeting of malignant B-cell clones. The table compares key features, including specificity, clinical evidence, toxicity profile, manufacturing complexity, and translational feasibility. CAR-T, chimeric antigen receptor T cells; CAAR-T, chimeric autoantibody receptor T cells; BCMA, B-cell maturation antigen; CRS, cytokine release syndrome; ICANS, immune effector cell-associated neurotoxicity syndrome; ICAHT, immune effector cell-associated hematotoxicity; MRD, minimal residual disease. Based on references (3–18, 23–27, 41–45).

## CAAR-T technology and mechanism of action

CAAR-T therapy is a conceptual modification of the CAR-T platform in which the extracellular recognition domain is designed to bind disease-associated immunoglobulin receptors rather than conventional lineage antigens (23–27). In autoimmune disease, CAAR constructs often display the relevant autoantigen extracellularly. B cells bearing autoreactive BCRs bind this autoantigen, thereby triggering CAAR-T activation and selective killing of the autoreactive clone.

The best-established example is pemphigus vulgaris, in which autoreactive B cells produce antibodies against desmoglein 3 (23). CAAR-T cells expressing desmoglein 3 can selectively recognize and eliminate desmoglein 3-specific B cells while sparing unrelated B cells (23). This model elegantly demonstrates the central principle of CAAR-T therapy: pathogenic B-cell populations can be targeted through the specificity of their BCR rather than through lineage markers.

In malignancy-directed CAAR-T therapy, the analogous target would be the tumor-specific idiotype expressed as part of the malignant BCR or surface immunoglobulin. Several engineering strategies could theoretically be used. A CAAR construct might contain an idiotype-binding domain, an antigen-mimetic structure, a peptide or protein ligand, or a patient-specific binding moiety selected against the malignant immunoglobulin variable region. Regardless of design, the construct would need to trigger T-cell activation upon binding to the malignant B-cell receptor and support cytotoxicity, proliferation, and persistence.

This approach imposes several technical requirements. First, the malignant immunoglobulin sequence must be identified accurately, typically through immunoglobulin gene sequencing or single-cell approaches (28–31, 41–45). Second, the target must be confirmed to be expressed on the malignant cell surface. Third, a binding domain must be designed and validated for specificity, affinity, and lack of cross-reactivity. Fourth, the final cellular product must demonstrate functional activity against patient-derived malignant cells or relevant models before clinical use. These steps are substantially more complex than targeting a shared antigen such as CD19.

## Preclinical and early clinical evidence for CAAR-T therapy

The feasibility of CAAR-T therapy has been demonstrated most convincingly in autoimmune disease models. In the seminal study by Ellebrecht and colleagues, CAAR-T cells engineered to express desmoglein 3 selectively eliminated autoreactive B cells in pemphigus vulgaris models while sparing non-pathogenic B cells (23). This work established proof-of-principle that engineered T cells can use BCR specificity to distinguish pathogenic from non-pathogenic B-cell populations.

Subsequent studies extended this concept to other antibody-mediated autoimmune contexts. GPIbα-directed CAAR-T cells have been evaluated in immune thrombocytopenia models, where they were designed to eliminate B cells producing pathogenic antibodies against platelet antigens (24). Other CAAR-T constructs have been developed to target autoreactive B cells producing antibodies against interferon-γ or other disease-associated antigens (25). Collectively, these studies support the biological feasibility of selective pathogenic B-cell depletion (23–27).

However, the relevance of these findings to cancer must be interpreted cautiously. Autoimmune disease models differ fundamentally from B-cell malignancies. The target population in autoimmunity may be smaller, less genetically unstable, less proliferative, and less capable of immune escape than a malignant clone. In cancer, the target cell population may be heterogeneous, spatially compartmentalized, immunosuppressive, and subject to strong selection pressure. Therefore, CAAR-T success in autoimmunity should be regarded as proof-of-mechanism rather than proof-of-antitumor efficacy.

At present, there is no robust clinical evidence establishing CAAR-T therapy as an effective treatment for B-cell malignancies (23–27, 40). No large randomized trials, pivotal studies, or approved malignancy-directed CAAR-T products exist. The application of CAAR-T to cancer therefore remains exploratory. This distinction is essential for a balanced interpretation of the field. Representative studies illustrating the development and application of CAAR-T therapy, primarily in autoimmune disease models, are summarized in **Table 3**.

**Table 3:** Representative CAAR-T studies and their relevance to B-cell malignancies.

Study/model	Disease context	CAAR target or recognition principle	Key finding	Relevance to malignancy-directed CAAR-T
Desmoglein 3 CAAR-T	Pemphigus vulgaris	Autoantigen-based recognition of autoreactive BCR	Selective elimination of autoreactive B cells	Establishes proof-of-principle for BCR-specific cellular targeting
GPIbα CAAR-T	Immune thrombocytopenia model	Platelet antigen-based recognition	Depletion of pathogenic B-cell populations in experimental systems	Supports feasibility of selective B-cell targeting beyond pemphigus
IFN-γ-directed CAAR-T	Autoantibody-mediated immune disease model	Recognition of B cells producing anti-IFN-γ antibodies	Selective elimination of disease-associated B cells	Demonstrates adaptability of CAAR design to different pathogenic BCR specificities
Idiotype-directed malignancy concept	B-cell malignancy models or proposed translational setting	Tumor-specific BCR/idiotype	Not clinically validated	Requires disease-specific preclinical validation before clinical translation

CAAR-T therapy has been primarily investigated in autoimmune disease models, where engineered T cells selectively eliminate autoreactive B-cell populations based on BCR specificity. The table summarizes key studies, their target antigens, experimental context, and relevance to malignancy-directed applications. Although these studies establish proof-of-mechanism, clinical evidence supporting CAAR-T in B-cell malignancies remains limited. CAAR-T, chimeric autoantibody receptor T cells; BCR, B-cell receptor. Based on representative studies and reviews (23–27).

## Disease-specific translational potential in hematologic malignancies

### Chronic lymphocytic leukemia

CLL represents one of the most biologically compelling contexts for idiotype-directed CAAR-T therapy. The disease is defined by clonal B cells expressing a characteristic BCR, and in a substantial fraction of patients, stereotyped BCR subsets suggest antigen-driven selection (29–31, 36). This creates a conceptual basis for targeting the malignant clone through its immunoglobulin identity. The clinical need is also relevant. Conventional CD19-directed CAR-T therapy has shown less consistent activity in CLL than in aggressive B-cell lymphoma or acute lymphoblastic leukemia (19–22). Multiple factors contribute to this limitation, including T-cell exhaustion, impaired immune synapse formation, suppressive cytokine networks, nurse-like cells, regulatory immune populations, and prior exposure to therapies that alter T-cell fitness (19–22). A more selective target alone may not overcome these barriers, but CAAR-T could theoretically reduce collateral B-cell depletion and provide a precision approach in selected patients.

The most rational CLL setting may not be bulky refractory disease but low-burden disease, molecular relapse, or minimal residual disease after debulking with targeted agents. In such contexts, lower tumor burden may reduce the inflammatory load and improve the feasibility of clone-specific eradication. However, CLL also illustrates a major challenge: the same immune dysfunction that limits CAR-T may impair CAAR-T expansion, persistence, and cytotoxicity. Therefore, CAAR-T development in CLL would require careful assessment of T-cell fitness, BCR stability, surface immunoglobulin density, and subclonal architecture (35–37).

### Follicular lymphoma and other indolent B-cell lymphomas

Indolent B-cell lymphomas are attractive from a therapeutic proportionality perspective. Many patients experience prolonged survival with repeated lines of therapy, and minimizing long-term immune damage is clinically meaningful. A strategy that selectively eliminates the malignant clone while preserving normal B-cell function could therefore be valuable.

Follicular lymphoma has a particularly strong historical connection to idiotype targeting. Patient-specific idiotype vaccines were among the earliest precision immunotherapy approaches in lymphoma (32–34). Although these vaccines did not become standard therapy, they demonstrated that the tumor immunoglobulin can serve as a disease-specific antigenic structure (32, 33). CAAR-T therapy could theoretically revisit this principle using an engineered cytotoxic effector platform rather than vaccine-induced immunity.

Nevertheless, several disease-specific limitations must be considered. Follicular lymphoma can display spatial and temporal heterogeneity, ongoing somatic hypermutation, clonal evolution, and transformation to aggressive lymphoma (35, 40). A single-idiotype CAAR-T product may be vulnerable to escape if the malignant population is not uniform. Therefore, idiotype-directed CAAR-T in follicular lymphoma may be most plausible in carefully selected patients with stable dominant clonotypes, low disease burden, or molecular persistence after standard therapy.

### Multiple myeloma

Multiple myeloma presents a more problematic setting for CAAR-T therapy. On one hand, monoclonal immunoglobulin production provides an easily identifiable patient-specific marker. On the other hand, the biological features of plasma-cell malignancy may undermine surface idiotype targeting. First, mature plasma cells often have reduced surface immunoglobulin expression compared with earlier B-cell stages. If the clonotypic immunoglobulin is predominantly secreted rather than membrane-bound, it may be a poor cellular target. Second, soluble antigen or paraprotein-related interference may theoretically reduce tumor-cell engagement, consistent with antigen-sink concerns described for BCMA-directed immune therapies (38). Third, multiple myeloma is frequently characterized by spatial heterogeneity, branching evolution, and multiple resistant subclones (40). These features may limit the durability of a highly specific idiotype-directed strategy.

For these reasons, CAAR-T is currently less compelling in active multiple myeloma than established targets such as BCMA, GPRC5D, or FcRH5 (12, 13, 38). A potential niche could exist in selected low-burden states, early plasma-cell disorders, or settings where surface immunoglobulin expression is demonstrable and soluble antigen interference is limited. However, this remains speculative and would require strong preclinical support.

### Minimal residual disease

Minimal residual disease may represent the most rational future setting for CAAR-T therapy in B-cell malignancies. In MRD, tumor burden is low, antigen load is reduced, and the goal is elimination of residual clones rather than rapid cytoreduction of bulky disease. This context may reduce the risks of antigen sink, severe inflammatory toxicity, and incomplete tissue penetration (38, 40). CAAR-T specificity could be particularly valuable if residual disease is molecularly well defined by a persistent clonotypic immunoglobulin sequence. In this setting, CAAR-T could theoretically function as a precision consolidation strategy after debulking with chemotherapy, targeted therapy, monoclonal antibodies, bispecific antibodies, or conventional CAR-T therapy. However, this application would require highly sensitive disease monitoring, longitudinal BCR sequencing, and evidence that the targeted idiotype remains stable at relapse.

## Manufacturing, economic feasibility, and regulatory barriers

**Figure 2:**
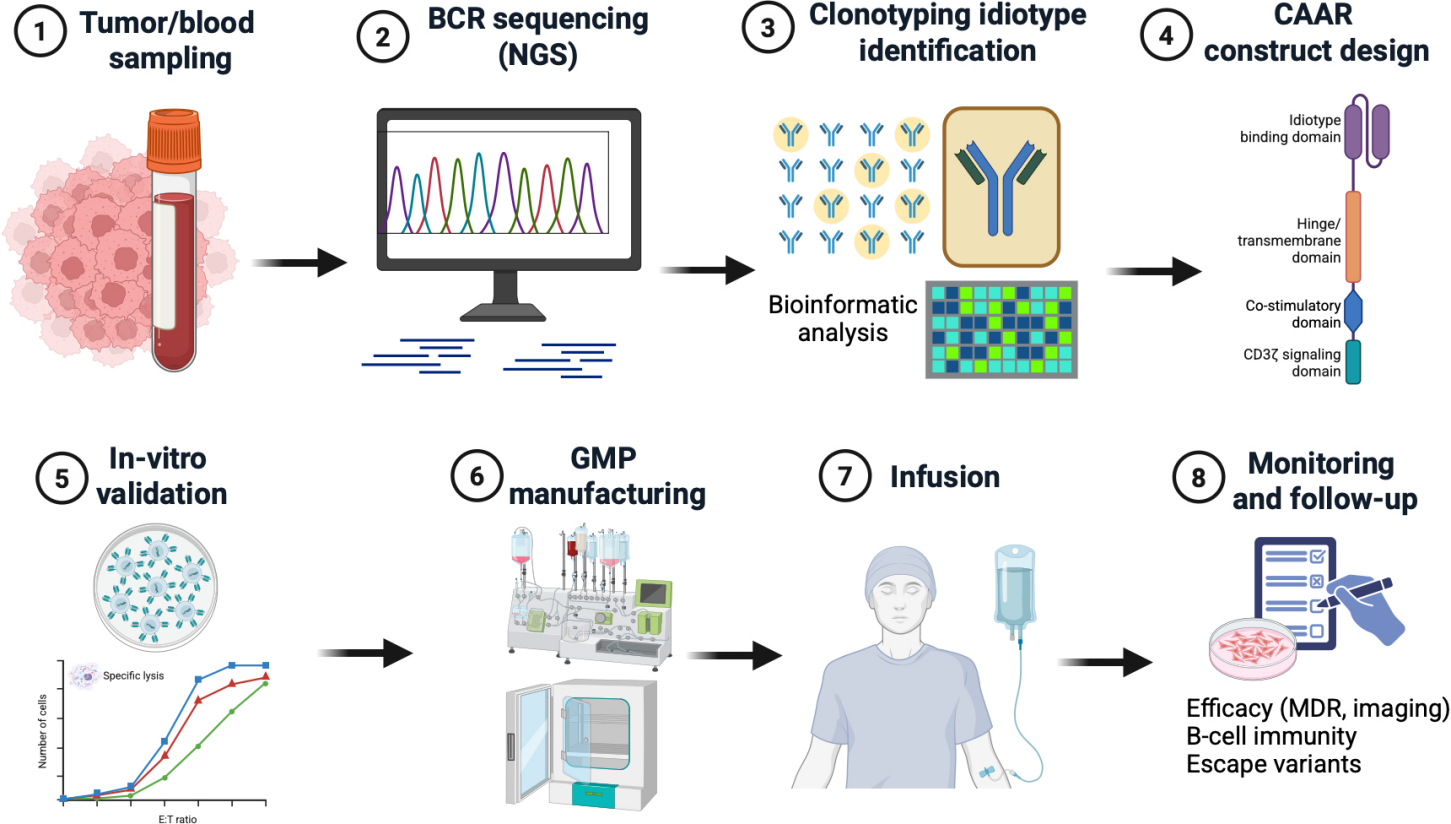
Proposed workflow for idiotype-directed CAAR-T development. The development of CAAR-T therapy involves multiple sequential steps, including tumor or blood sampling, B-cell receptor (BCR) sequencing, identification of clonotypic immunoglobulin targets, design of a CAAR construct, functional validation, and GMP-compliant manufacturing prior to clinical administration (28–31, 41–45). Each step introduces specific technical and translational challenges, including target heterogeneity, antigen stability, manufacturing complexity, and regulatory considerations (35, 40–45). This workflow highlights the individualized nature of CAAR-T therapy and underscores key barriers to its scalability and clinical implementation. Created in BioRender.com by Buczek, T. (2026) https://BioRender.com/30zejlw.

The greatest practical barrier to malignancy-directed CAAR-T therapy is individualization. Conventional CAR-T products target shared antigens, allowing development of standardized constructs applicable to broad patient populations (41–45). Idiotype-directed CAAR-T would likely require patient-specific target identification and receptor design, creating a substantially more complex production model (41–45). The development of CAAR-T therapy requires a series of coordinated steps from target identification to clinical administration. This workflow is summarized in **Figure 2**.

A potential CAAR-T workflow would include tumor sampling, immunoglobulin/BCR sequencing, identification of the dominant clonotypic target, confirmation of surface expression, design or selection of an idiotype-binding recognition domain, functional validation, vector production or construct assembly, T-cell manufacturing, quality control testing, lymphodepletion, infusion, and post-infusion monitoring. Each step introduces time, cost, and failure risk. Economic feasibility is therefore uncertain. CAR-T therapy is already among the most expensive cancer treatments, despite targeting shared antigens and using increasingly standardized manufacturing platforms (43–45). CAAR-T could be more expensive if each product requires individualized molecular design and validation. Cost drivers would include sequencing, bioinformatic analysis, custom construct development, GMP-grade production, potency assays, release testing, and potential delays requiring bridging therapy (41–45).

Scalability is another major concern. A therapy that is scientifically elegant but impossible to deliver within clinically acceptable timeframes will have limited impact. For aggressive or rapidly progressive disease, individualized CAAR-T manufacturing may be too slow unless automated and modular platforms are developed (41–45). For indolent disease or MRD, longer production timelines may be more acceptable, making these settings more realistic for early development.

Regulatory challenges are equally important (41–45). If each CAAR-T product is patient-specific, defining comparability, potency, safety, and release criteria becomes difficult. Regulators may need to determine whether each construct is a unique product or part of a platform technology. Batch-to-batch consistency, off-target binding, insertional safety, receptor specificity, and functional potency would all require standardized assessment. Personalized receptor design also raises questions about how much preclinical validation is necessary for each patient-specific construct.

Potential solutions include modular receptor platforms, libraries of validated idiotype-binding domains, rapid non-viral manufacturing, automated closed-system production, standardized sequencing pipelines, and adaptive regulatory frameworks. However, these remain developmental solutions rather than current clinical realities. The main translational steps required for CAAR-T development, together with their associated challenges and potential solutions, are outlined in **Table 4**.

**Table 4:** Translational feasibility barriers for idiotype-directed CAAR-T therapy.

Step	Key requirement	Main barrier	Potential solution
Target identification	Accurate BCR/Ig sequencing	Tumor heterogeneity; sample quality	Single-cell sequencing; longitudinal sampling
Target validation	Confirmation of surface expression	Low or variable surface Ig	Flow cytometry; antigen-density thresholds
Binding-domain design	Specific recognition of tumor idiotype	Cross-reactivity; time-consuming development	Modular libraries; computational design
Functional testing	Demonstration of cytotoxicity and specificity	Lack of standardized assays	Patient-derived models; potency assay harmonization
Manufacturing	GMP production of individualized product	Cost, time, batch failure	Automated closed systems; rapid manufacturing
Regulatory approval	Consistent safety and potency framework	Each product may be unique	Platform-based regulatory strategy
Clinical implementation	Timely delivery to appropriate patients	Disease progression during production	MRD/low-burden indications; bridging therapy

The development of CAAR-T therapy requires multiple sequential steps, including target identification, validation of clonotypic immunoglobulin expression, receptor design, functional testing, and GMP-compliant manufacturing. Each step introduces specific technical, economic, and regulatory challenges. The table summarizes these barriers and outlines potential strategies to improve feasibility and scalability. CAAR-T, chimeric autoantibody receptor T cells; BCR, B-cell receptor; GMP, Good Manufacturing Practice. Based on manufacturing and scalability considerations discussed in references (41–45).

## Potential clinical positioning of CAAR-T therapy

CAAR-T therapy is unlikely to replace conventional CAR-T therapy in indications where approved products are already effective, available, and supported by mature evidence. In aggressive lymphoma, acute lymphoblastic leukemia, and multiple myeloma, current CAR-T approaches have clear clinical utility (5–13). CAAR-T would need to demonstrate a major advantage in efficacy, safety, durability, or immune preservation to justify its complexity. A more realistic role may be in narrowly defined clinical niches. These could include diseases with stable clonotypic BCR expression, low tumor burden, high need for immune preservation, or limited benefit from conventional CAR-T therapy (19–22, 28–40). CLL and indolent lymphomas may therefore be more plausible early targets than active multiple myeloma. MRD-directed therapy may be especially attractive because the biological and logistical challenges are less severe than in bulky refractory disease. Combination strategies may also be important. CAAR-T could be paired with agents that reduce tumor burden, improve T-cell fitness, or suppress immune escape. Examples might include BTK inhibitors in CLL, anti-CD20 antibodies in lymphoma, immune checkpoint modulation in selected settings, or sequential use after conventional therapies. Dual-target or multi-specific designs may reduce escape risk by combining idiotype recognition with broader B-cell or tumor-associated antigen targeting (35, 39, 40, 42). However, increasing target breadth may also reduce the immune-sparing advantage that makes CAAR-T attractive.

## Discussion

CAAR-T therapy represents a conceptually compelling extension of cellular immunotherapy, aiming to increase targeting precision by shifting from shared lineage antigens to clonotypic immunoglobulin structures. This approach directly addresses one of the central limitations of conventional CAR-T therapy, namely on-target, off-tumor toxicity resulting from depletion of the normal B-cell compartment. In principle, idiotype-directed targeting could enable selective elimination of malignant clones while preserving non-malignant B cells, thereby maintaining humoral immunity and reducing long-term treatment-related morbidity (14, 15).

However, this increased specificity is inherently accompanied by biological trade-offs. Unlike lineage antigens such as CD19 or BCMA, clonotypic targets are individualized and may not be uniformly expressed across all malignant cells. B-cell malignancies are dynamic systems characterized by clonal evolution, subclonal diversity, and, in some entities, ongoing somatic hypermutation. Under these conditions, selective pressure exerted by a highly specific CAAR-T product may promote the emergence or expansion of antigen-negative variants (35, 39, 40). This phenomenon has been previously observed in idiotype-directed therapeutic approaches and highlights immune escape as a fundamental challenge rather than a secondary consideration (39).

Another critical limitation is the current evidence gap. Most available data on CAAR-T therapy originate from autoimmune disease models, where the target population is typically less heterogeneous, less proliferative, and less capable of immune evasion than malignant clones (23–27). While these studies provide strong proof-of-mechanism for selective B-cell targeting, they do not directly translate into evidence of antitumor efficacy. Cancer-specific validation, including preclinical models that capture clonal heterogeneity and tumor microenvironment complexity, remains largely absent (35, 40). As a result, the application of CAAR-T in B-cell malignancies should currently be regarded as exploratory.

The feasibility of CAAR-T is likely to be strongly disease-dependent. In chronic lymphocytic leukemia, the presence of clonotypic and, in some cases, stereotyped B-cell receptors provides a biologically coherent target. At the same time, the well-described dysfunction of T cells in CLL and the immunosuppressive tumor microenvironment may limit the effectiveness of any autologous cellular therapy, including CAAR-T (19–22). In indolent lymphomas, the relatively long disease course and the need to minimize cumulative toxicity make selective targeting strategies particularly attractive. However, intratumoral heterogeneity and the risk of transformation must be considered when evaluating the durability of a clone-specific approach. In multiple myeloma, the applicability of CAAR-T appears more limited due to reduced surface immunoglobulin expression and high levels of circulating paraprotein, which may act as an antigen sink and impair receptor engagement (38).

Among potential clinical scenarios, minimal residual disease (MRD) represents a particularly attractive setting for CAAR-T development. Lower tumor burden may reduce the impact of antigen sink, clonal diversity, and inflammatory toxicity, while allowing highly specific targeting of residual malignant clones. In this context, CAAR-T could function as a precision consolidation strategy following cytoreductive therapy rather than as a primary treatment modality for advanced disease. This hypothesis aligns with the broader trend toward MRD-directed therapeutic interventions but requires prospective validation.

Beyond biological considerations, translational feasibility represents a major barrier. The individualized nature of CAAR-T implies a need for patient-specific target identification, receptor design, and validation, which significantly increases manufacturing complexity, cost, and turnaround time (41–45). Unlike conventional CAR-T therapies targeting shared antigens, CAAR-T may not benefit from standardized production pipelines in its current conceptual form. In addition, regulatory frameworks for highly individualized cellular products remain underdeveloped, particularly with respect to potency assays, product comparability, and quality control for unique constructs. These factors collectively raise concerns regarding scalability and real-world implementation.

Future development of CAAR-T will likely depend on integration with emerging technologies. Advances in single-cell sequencing, computational protein design, and synthetic biology may facilitate rapid identification and targeting of clonotypic immunoglobulins (41–45). Modular or multi-specific receptor platforms could mitigate the risk of antigen escape, although potentially at the cost of reduced specificity. Combination strategies aimed at improving T-cell fitness or reducing tumor burden prior to CAAR-T infusion may also enhance efficacy. However, such approaches must balance complexity with clinical feasibility.

## Conclusions

CAAR-T therapy introduces a conceptually distinct approach to cellular immunotherapy by targeting clonotypic immunoglobulin structures rather than shared lineage antigens. This strategy has the potential to increase specificity and reduce the immune-related toxicity associated with conventional CAR-T therapies. However, its application in B-cell malignancies remains largely hypothetical and is not supported by robust clinical evidence (23–27).

Several key challenges must be addressed before CAAR-T can be considered a viable therapeutic option. These include biological limitations such as clonal heterogeneity, antigen instability, and immune escape (35, 39, 40), as well as technical and translational barriers related to individualized receptor design, manufacturing complexity, cost, and regulatory uncertainty (41–45). Importantly, the majority of current data supporting CAAR-T originate from autoimmune disease models and cannot be directly extrapolated to cancer. The most plausible future applications of CAAR-T may lie in carefully selected clinical contexts, particularly in diseases characterized by stable clonotypic targets and in settings of low tumor burden, such as minimal residual disease. In these scenarios, the balance between specificity and feasibility may be more favorable.

Overall, CAAR-T should be viewed as a promising but early-stage precision immunotherapy platform. Its successful translation into clinical practice will depend on rigorous disease-specific validation, technological advances enabling scalable production, and the demonstration of meaningful clinical benefit in appropriately selected patient populations.
